# Enhanced acid-stress tolerance in *Lactococcus lactis* NZ9000 by overexpression of ABC transporters

**DOI:** 10.1186/s12934-019-1188-8

**Published:** 2019-08-13

**Authors:** Zhengming Zhu, Jinhua Yang, Peishan Yang, Zhimeng Wu, Juan Zhang, Guocheng Du

**Affiliations:** 1 Key Laboratory of Industrial Biotechnology, Ministry of Education, School of Biotechnology, Jiangnan University, 1800 Lihu Road, Wuxi, 214122 Jiangsu China; 2 The Key Laboratory of Carbohydrate Chemistry and Biotechnology, Ministry of Education, Jiangnan University, 1800 Lihu Road, Wuxi, 214122 Jiangsu China; 3 School of Biotechnology, Jiangnan University, 1800 Lihu Road, Wuxi, 214122 Jiangsu China

**Keywords:** *Lactococcus lactis* NZ9000, ABC transporters, Acid-stress tolerance, Anti-acid components, Transcriptomics

## Abstract

**Background:**

Microbial cell factories are widely used in the production of acidic products such as organic acids and amino acids. However, the metabolic activity of microbial cells and their production efficiency are severely inhibited with the accumulation of intracellular acidic metabolites. Therefore, it remains a key issue to enhance the acid tolerance of microbial cells. In this study, we investigated the effects of four ATP-binding cassette (ABC) transporters on acid stress tolerance in *Lactococcus lactis*.

**Results:**

Overexpressing the *rbsA*, *rbsB*, *msmK*, and *dppA* genes exhibited 5.8-, 12.2-, 213.7-, and 5.2-fold higher survival rates than the control strain, respectively, after acid shock for 3 h at pH 4.0. Subsequently, transcriptional profile alterations in recombinant strains were analyzed during acid stress. The differentially expressed genes associated with cold-shock proteins (*csp*), fatty acid biosynthesis (*fabH*), and coenzyme A biosynthesis (*coaD*) were up-regulated in the four recombinant strains during acid stress. Additionally, some genes were differentially expressed in specific recombinant strains. For example, in *L. lactis* (RbsB), genes involved in the pyrimidine biosynthetic pathway (*pyrCBDEK*) and glycine or betaine transport process (*busAA* and *busAB*) were up-regulated during acid stress, and the *argG* genes showed up-regulations in *L. lactis* (MsmK). Finally, we found that overexpression of the ABC transporters RbsB and MsmK increased intracellular ATP concentrations to protect cells against acidic damage in the initial stage of acid stress. Furthermore, *L. lactis* (MsmK) consistently maintained elevated ATP concentrations under acid stress.

**Conclusions:**

This study elucidates the common and specific mechanisms underlying improved acid tolerance by manipulating ABC transporters and provides a further understanding of the role of ABC transporters in acid-stress tolerance.

**Electronic supplementary material:**

The online version of this article (10.1186/s12934-019-1188-8) contains supplementary material, which is available to authorized users.

## Background

As a microbial cell factory, *Lactococcus lactis* is a highly useful bacterial species that is capable of producing chemicals, including lactic acid and vitamins, and is used for fermented foods. It shows stable fermentation performance and phage resistance, and contributes to flavor development [1]. Furthermore, *L. lactis* is often used for genetic engineering due to its rapid growth, clear genetic background and abundant bioinformatics resources [2]. The rapid development of food-grade expression systems represented by sugar and nisin induction has expanded the applications of *L. lactis* in food processing [3, 4]. However, during industrial fermentation and food processing, *L. lactis* is frequently confronted with various stresses conditions including oxidative, bile salt, and cold stresses, especially acid stress because of the accumulation of lactate and other acidic metabolites [5, 6]. The decrease in pH values affects the growth and metabolic activity of cells, thereby reducing the production efficiency of the food and affecting the prebiotic functions [7]. Thus, enhancing the acid-stress tolerance of *L. lactis* can contribute to the production of high-quality fermented foods.

Several strategies have been proposed to increase the acid-stress tolerance of bacterial strains. Evolutionary engineering strategies are extensively used to improve the acid tolerance of microbial cells [8]. The acid tolerance of *Lactobacillus casei* Zhang has been shown to be increased by adaptive evolution, and the evolved mutant exhibited a 318-fold higher survival rate than that of the parent strain at pH 3.3 for 3 h [9]. Notably, genome shuffling is an effective method to improve the acid tolerance of *Lactobacillus* spp. and to facilitate the evolution of *Lactobacillus* populations [10]. In addition, global transcription machinery engineering (gTME) can improve cellular phenotypes, especially in terms of cellular tolerance [11]. Moreover, based on biochemical engineering strategies, the exogenous addition of various protective agents could help microbial cells against acid stress. For example, aspartate has been found to protect *L. casei* against acid stress [12]. Recently, the development of systems biology has accelerated our understanding of mechanisms underlying improved acid tolerance [13]. Based on this novel method, various anti-acid components have been identified, and reverse metabolic engineering approaches have been employed to improve acid resistance.

A series of anti-acid components has been found to contribute to acid-stress tolerance. These anti-acid components mainly include genes acting as regulatory factors, molecular chaperone proteins, non-coding sRNAs, sigma factors and transport (membrane) proteins [14–18]. Moreover, to maintain the equilibrium conditions necessary for cell survival under acid stress, the transport of various substrates including sugars, peptides, amino acids, ions, and vitamins is required, which is accomplished by transporters present on the cell membrane. Of all the transport proteins, ABC transporters comprise one of the largest protein superfamilies, and they are known to mediate the transport of various substrates across membranes [19]. These transporters power the transport of a variety of substrates across membranes through the binding and hydrolysis of ATP. The ABC transporter is composed of two transmembrane domains (TMD) and two nucleotide-binding domains (NBD) [20]. Various transporters have been illustrated to contribute to stress tolerance. Wang et al. found that oligopeptide transporter substrate-binding protein (OppA) could help to improve bile-, heat- and salt-stress tolerance in *Lactobacillus salivarius* Ren [21]. In addition, the *thiT* gene, encoding thiamine uptake system, has been found to be necessary for full acid tolerance in *Listeria monocytogenes*; a *thiT* mutant strain resulted in significantly higher acid sensitivity than the control strain [22]. In *Saccharomyces cerevisiae*, the deletion of *ADY2* gene, encoding an acetate transporter, resulted in enhanced acetic acid and hydrogen peroxide tolerance [23].

In our previous study, three acid-tolerant strains were acquired using genome mutagenesis combined with high-throughput technology. Then, several anti-acid components were identified based on the comparative transcriptomics analysis of parent and mutant strains. However, among these potential targets, ABC transporters have still not been explored. It will be interesting to examine the roles of these transporters in acid tolerance in *Lactococcus* species. In this study, we first investigated the effect of four ABC transporters on acid tolerance. Subsequently, comparative transcriptomics analysis was performed to further investigate the mechanisms underlying improved acid tolerance by manipulating ABC transporters.

## Materials and methods

### Bacterial strains, plasmids, and culture conditions

All the bacterial strains and plasmids used in this study are listed in Table 1. *L. lactis* NZ9000 and *E. coli* MC1061 were used throughout this study. *L. lactis* cells were grown in GM17 medium (M17 broth supplied with 0.5% glucose) at 30 °C without shaking (Oxoid M17 broth; Thermo Fisher Scientific, Waltham, MA, USA). *E. coli* MC1061 was used as the host for plasmid construction. *E. coli* was incubated in LB (Luria–Bertani) medium at 37 °C with shaking at 220 rpm. Media were supplemented with chloramphenicol for the selection at concentrations of 100 μg/ml for *E. coli* and 5 μg/ml for *L. lactis*.

**Table 1:** Strains and plasmids used in this study

Strains or plasmids	Relevant property^a^	Reference or source
Strains		
*L*. *lactis* ssp. cremoris NZ9000	MG1363 *pepN*:*:nisRK*	[45]
*L. lactis* (Vector)	*L. lactis* containing pNZ8148/Vector, Cm^r^	[25]
*L. lactis* (RbsA)	*L. lactis* containing pNZ8148/RbsA, Cm^r^	This study
*L. lactis* (RbsB)	*L. lactis* containing pNZ8148/RbsB, Cm^r^	This study
*L. lactis* (MsmK)	*L. lactis* containing pNZ8148/MsmK, Cm^r^	This study
*L. lactis* (DppA)	*L. lactis* containing pNZ8148/DppA, Cm^r^	This study
*E. coli* MC1061	araD139, Δ(ara, leu)7697, ΔlacX74, galU-, galK-, hsr-, hsm^+^, strA	[46]
Plasmids		
pNZ8148	Cm^r^; inducible expression vector with nisA promoter	[47]
pNZ8148/RbsA	Cm^r^; pNZ8148 derivative containing a *rbsA* gene	This study
pNZ8148/RbsB	Cm^r^; pNZ8148 derivative containing a *rbsB* gene	This study
pNZ8148/MsmK	Cm^r^; pNZ8148 derivative containing a *msmK* gene	This study
pNZ8148/DppA	Cm^r^; pNZ8148 derivative containing an d*ppA* gene	This study

^a^Cm^r^, chloramphenicol resistant

### Cloning and overexpression of ABC transporters

The *rbsA*, *rbsB*, *msmK* and *dppA* genes were amplified using *L. lactis* NZ9000 genomic DNA as a template, and the *Nco*I and *Hin*dIII (or *Xba*I) restriction sites were simultaneously inserted into the amplified gene fragments. The resulting fragments were digested with *Nco*I and *Hin*dIII (or *Xba*I) and subsequently ligated into plasmid pNZ8148, which was digested with the corresponding restriction enzymes. The ligated products were introduced into *Escherichia coli* MC1061, then positive clones were selected through colony PCR, followed by Sanger sequencing. The recombinant plasmids were named pNZ8148/RbsA, pNZ8148/RbsB, pNZ8148/MsmK, and pNZ8148/DppA, respectively, and subsequently introduced into *L. lactis* NZ9000 by electroporation [24]. The resulting strains were named *L. lactis* (RbsA), *L. lactis* (RbsB), *L. lactis* (MsmK) and *L. lactis* (DppA), respectively. An empty pNZ8148 plasmid was also transformed into *L. lactis* NZ9000 to construct the recombinant strain *L. lactis* (Vector) as a control. All primers used in this study are listed in Additional file 1: Table S1.

### Acid-stress tolerance assays

To measure *L. lactis* acid tolerance, the cells were induced at OD_600_ of 0.5 by adding 10 ng/ml nisin, then cultured for 6 h (exponential phase). The induced cells were harvested and washed twice with 0.85% saline solution, then resuspended in an equal volume of acidic GM17 medium (adjusted to pH 4.0 with lactic acid) with 10 ng/ml nisin and 10 μg/ml chloramphenicol. Cell viability was determined at various time points by counting the number of colonies after 10 µl of serially diluted cell suspension was spotted on GM17 agar plates containing 10 μg/ml chloramphenicol and cultured at 30 °C for 24 h [25]. Each sample was performed in triplicate, and colonies containing between 20 and 200 CFU were counted.

### RNA-Seq sample preparation and transcriptome analysis

After the induced cells reached the exponential phase, an aliquot was harvested from the culture and used as the unstressed group (0 h acid treatment). Meanwhile, the remaining equal volume of culture was subjected to acid stress (pH 4.0, adjusted with lactic acid) for 2.5 h, followed by collection by centrifugation at 8000*g* for 4 min at 4 °C and washing twice with ice-cold 50 mM phosphate-buffered saline (PBS). The pellets were quickly placed in liquid nitrogen to quench cellular metabolism, and the total RNA was extracted by using the RNAprep pure bacteria kit (Tiangen, Beijing, China) according to the manufacturer’s protocol. Purified RNA was quantified using the NanoDrop ND-2000 apparatus (Thermo Fisher Scientific, Waltham, MA, USA). RNA samples were stored at − 80 °C until transcriptome analysis.

Samples were sent to Vazyme Biotech. (Nanjing, China) for transcriptome sequencing. rRNA removal, mRNA purification and fragmentation, cDNA synthesis, adapter ligation, and PCR amplification were performed to construct a cDNA library. Library quantification was examined using an Agilent 2100 bioanalyzer (Agilent Technologies, Santa Clara, CA, USA). Sequencing was performed on an Illumina HiSeq 2500 system (Illumina, San Diego, CA, USA).

The base composition of raw reads and quality distribution of the bases along the reads were analyzed to perform quality control. Then, the raw reads were filtered into clean reads and aligned to the reference sequences using HISAT2 [26]. Transcript assembly and the calculation of gene-expression levels were performed using StringTie [27]. Analysis of differentially expressed genes (DEGs) was performed using DEGseq [28]. The significance of differences in gene expression was defined as p < 0.05 and fold changes ≥ 2. The Gene Ontology (GO) analysis was performed with the phyper (Hypergeometric test) using the GO database (http://www.geneontology.org/).

### Determination of intracellular ATP concentration

The induced cells (at 6 h) were subjected to acid stress (pH 4.0, adjusted with lactic acid) and then sampled at various time points (0, 1, and 2.5 h). Cellular metabolism was quenched using liquid nitrogen, then cells were harvested by centrifugation at 10,000*g* for 10 min at 4 °C. The intracellular ATP concentration was measured by using an ATP assay kit (Beyotime, Shanghai, China). The prote in concentration of each sample was measured with a bicinchoninic acid (BCA) protein assay kit (Tiangen, Beijing, China) using bovine serum albumin as a standard. The final ATP concentration was expressed as nmol/mg protein.

## Results

### Overexpression of ABC transporters improves acid-stress tolerance of *L. lactis*

**Fig. 1:**
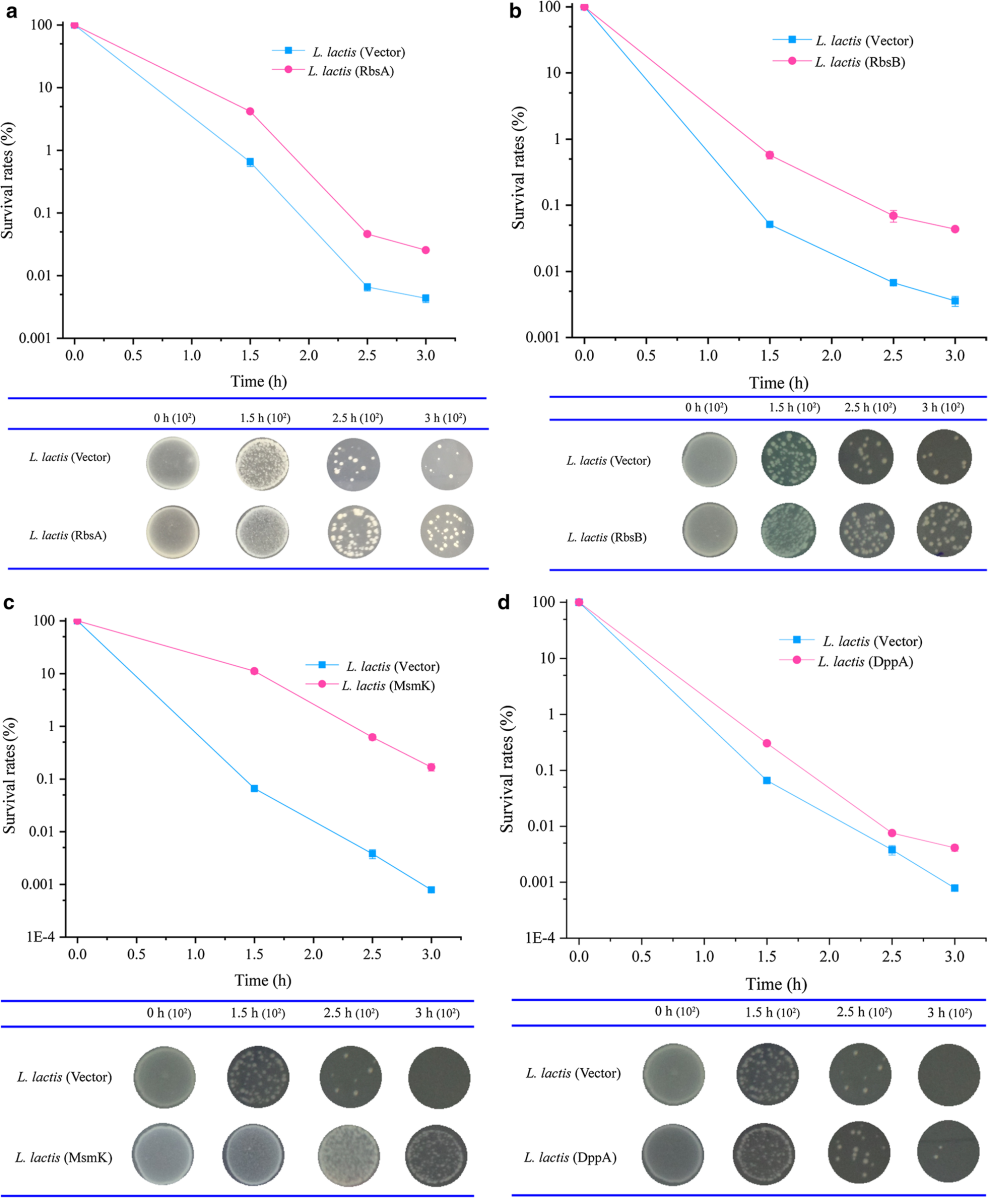
The survival rates of the control and recombinant strains under acid-stress conditions. **a** *L. lactis* (RbsA); **b** *L. lactis* (RbsB); **c** *L. lactis* (MsmK); **d** *L. lactis* (DppA). Error bars represent the mean ± standard deviation of three replicates

To evaluate the acid stress tolerance of the ABC transporters, four genes were overexpressed in *L. lactis* NZ9000 (Table 2). Then, their survival rates were determined to clarify the effects of these recombinant strains on acid tolerance. The four recombinant strains exhibited higher survival rates after acid stress at various time points (Fig. 1). After acid shock for 2.5 h, the recombinant strains *L. lactis* (RbsA), *L. lactis* (RbsB), *L. lactis* (MsmK), and *L. lactis* (DppA) exhibited 7.0-, 10.3-, 163.3-, and 2.0-fold higher survival rates than the control strain, respectively. Moreover, after acid shock for 3 h, the survival rates of recombinant strains were markedly higher than that of the control strain (5.8-, 12.2-, 213.7-, and 5.2-fold, respectively) (Fig. 1). Based on these results, we can conclude that overexpression of the four ABC transporters can confer acid stress tolerance on *L. lactis*.

**Table 2:** Characteristics of ABC transporters

Gene name	Gene ID	Gene length (bp)	Product
*rbsA*	LLNZ_RS04075	1479	d-ribose ABC transporter ATP-binding protein
*rbsB*	LLNZ_RS04085	975	d-ribose ABC transporter substrate-binding protein
*msmK*	LLNZ_RS02280	1137	Sugar ABC transporter ATP-binding protein
*dppA*	LLNZ_RS01875	1653	Oligopeptide ABC transporter substrate-binding protein

### Overall gene expression profiles in response to acid stress

Due to the remarkable improvement in the acid stress tolerance of recombinant strains, we further investigated the possible mechanisms underlying improved acid tolerance mediated by the ABC transporters. Thus, transcriptome sequencing was performed to compare different gene-expression profiles between the control and recombinant strains at 0 and 2.5 h.

For transcriptomic analysis, differential expression was set at a threshold of p < 0.05 and fold change ≥ 2. A total of 30 and 33 DEGs were identified between the recombinant strain *L. lactis* (RbsA) and control strain *L. lactis* (Vector) at 0 and 2.5 h, respectively (Additional file 1: Fig. S1a and Table S2). For *L. lactis* (RbsB), 157 and 146 DEGs were identified compared to the control strain at 0 and 2.5 h, respectively (Additional file 1: Fig. S1b and Table S2). In addition, 44 and 33 DEGs were identified between strain *L. lactis* (MsmK) and *L. lactis* (Vector) at 0 and 2.5 h, respectively (Additional file 1: Fig. S1c and Table S2). Finally, compared to the control strain, there were 43 and 44 DEGs in *L. lactis* (DppA) at 0 and 2.5 h, respectively (Additional file 1: Fig. S1d and Table S2).

Subsequently, GO analysis was performed to determine significantly differentially expressed gene clusters. We found here that the main changes in response to acid stress occurred among regulation of biological process, the establishment of localization, and small molecular metabolic process under normal condition (0 h). In addition, GO groups involved in isomerase activity, regulation of biological process, and small molecular metabolic process were significantly affected by acid stress (2.5 h) (Additional file 1: Fig. S1e).

### Transcriptome analysis of the RbsA, RbsB, MsmK, and DppA-overexpressing strain

**Fig. 2:**
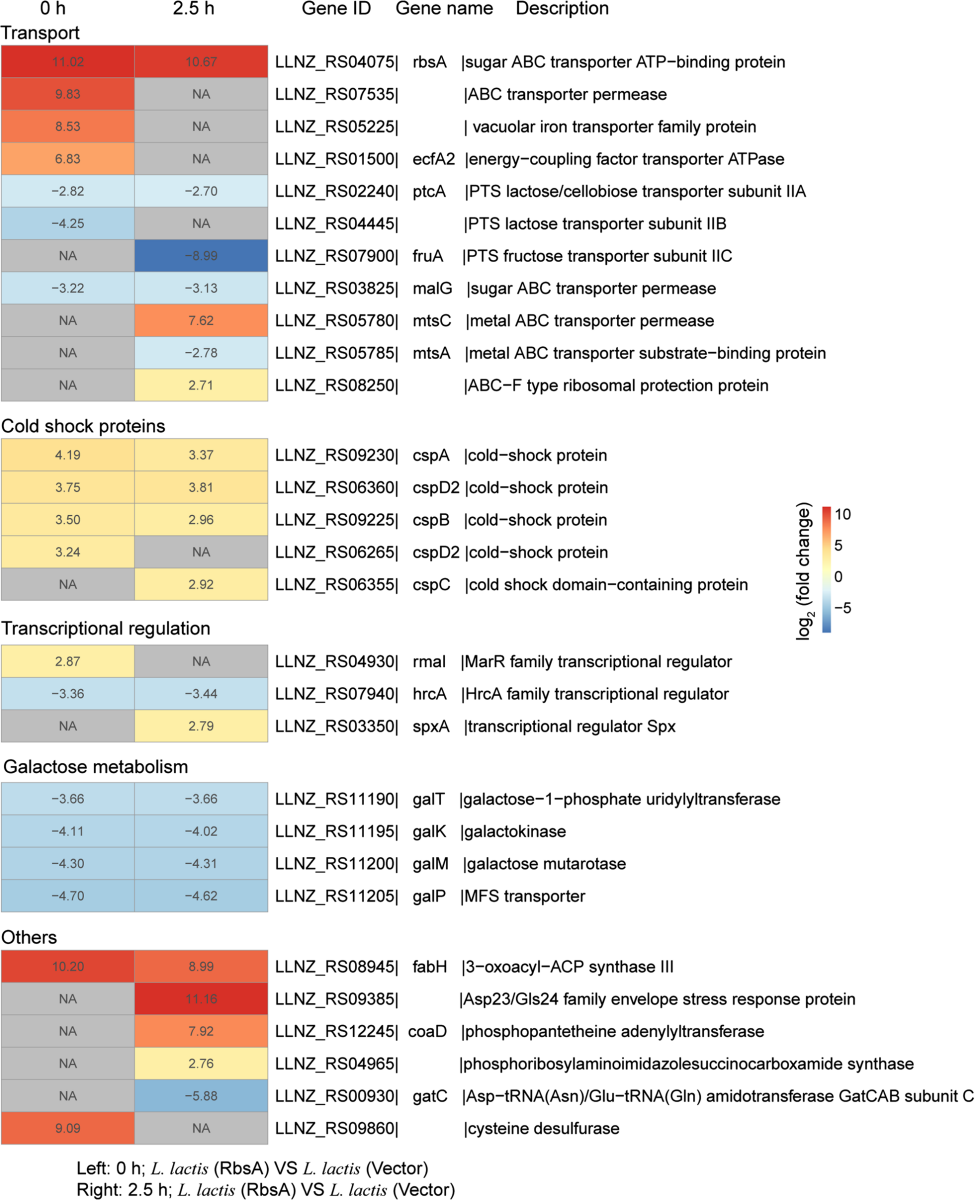
Heatmap of important differentially expressed genes in the recombinant strain [*L. lactis* (RbsA)] relative to the control strain [*L. lactis* (Vector)] under normal (0 h) and acid-stress (2.5 h) conditions. Each gene shows the expression ratio (log_2_-fold change). NA represents the expression of gene was upregulated or downregulated with a less than twofold change. Genes with at least a twofold change are shown. Adjusted p < 0.05 for all data selected

Based on the GO analysis, various biological processes including transport, metabolism, and transcriptional regulation were shown to be affected by acid stress. Thus, we analyzed the key DEGs involved in these biological processes. In *L. lactis* (RbsA), we found that the *rbsA* gene showed dramatic 11.02- and 10.67-fold (log_2_ (fold change)) up-regulations, respectively, under normal and acid-stress conditions (Fig. 2). Three genes related to transport (*LLNZ_RS07535*, *LLNZ_RS05225*, and *ecfA2*) were highly up-regulated under normal conditions, and the genes *LLNZ_RS08250* and *mtsC* increased 7.62- and 2.71-fold, respectively, during acid stress. In addition, the *cspABD2* genes, which encode cold-shock proteins, were consistently up-regulated under normal and acid-stress conditions. However, genes associated with galactose metabolism (*galKMPT*) were down-regulated under both conditions. Moreover, the transcriptional regulator *rmal* was up-regulated under normal conditions, while the regulator *spxA* was up-regulated during acid stress. Interestingly, the gene *fabH* (3-oxoacyl-ACP synthase III), which involves in fatty acid biosynthesis pathway, showed dramatic 10.20- and 8.99-fold upregulations, respectively, under both conditions. We also found that the genes *LLNZ_RS09385* (Asp23/Gls24 family envelope stress response protein), *coaD* (phosphopantetheine adenylyltransferase), and *LLNZ_RS04965* (phosphoribosylaminoimidazole-succinocarboxamide synthase) were up-regulated in the recombinant strain during acid stress.

**Fig. 3:**
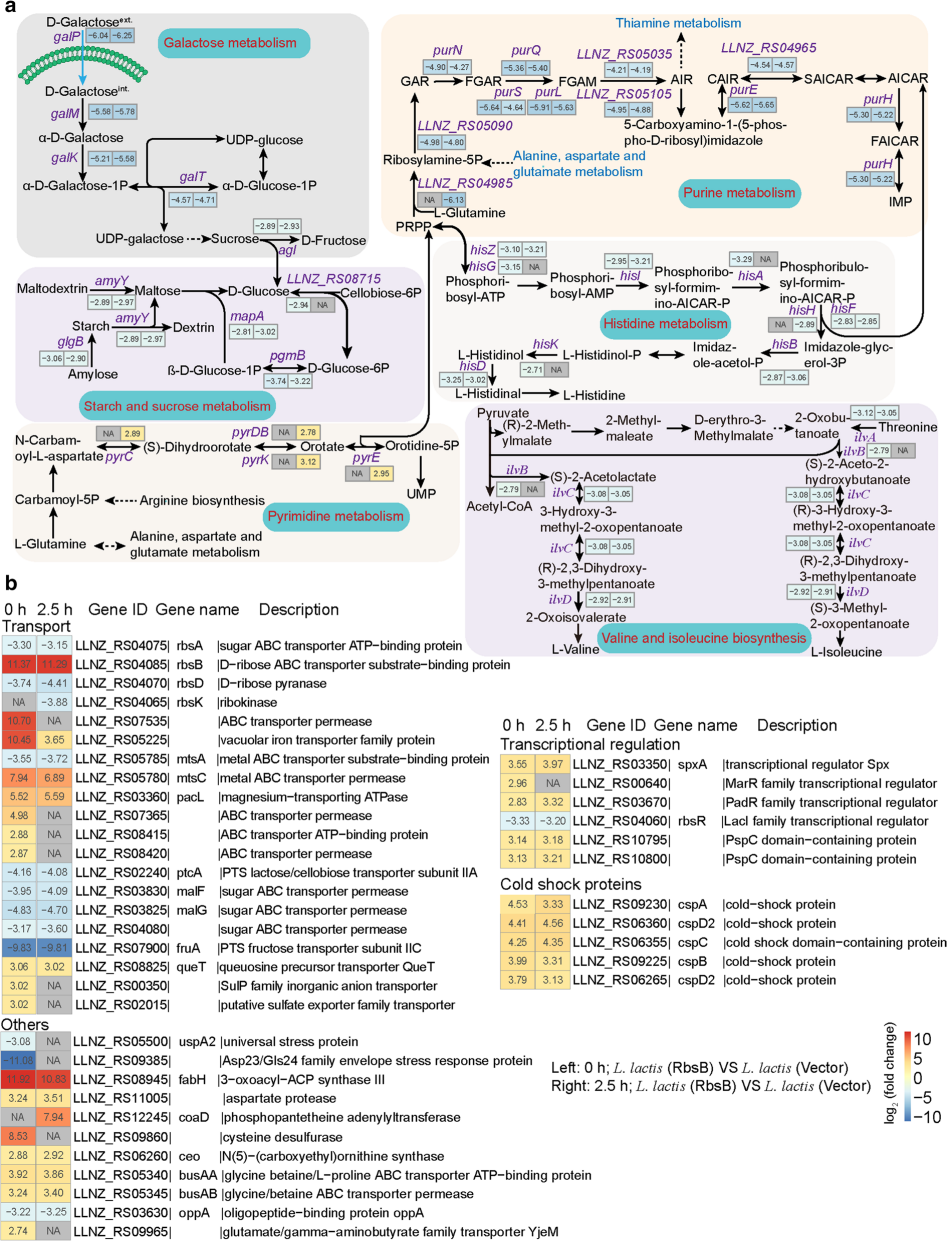
Important differentially expressed genes in the recombinant strain (*L. lactis* (RbsB)) relative to the control strain (*L. lactis* (Vector)) under normal (0 h) and acid-stress (2.5 h) conditions. **a** Differentially expressed genes involved in the galactose metabolism, starch and sucrose metabolism, pyrimidine metabolism, purine metabolism, histidine metabolism, and valine and isoleucine biosynthesis. **b** Heatmap of differentially expressed genes involved in another biological process. Each gene shows the expression ratio (log_2_-fold change). NA represents the expression of gene was upregulated or downregulated with a less than twofold change. Genes with at least a twofold change are shown. Adjusted p < 0.05 for all data selected

Next, we found here that five genes related to transport (*rbsB*, *LLNZ_RS05225*, *mtsC*, *pacL*, and *queT*) were highly up-regulated in *L. lactis* (RbsB) under normal and acid-stress conditions. Among these genes, the *rbsB* gene exhibited dramatic 11.37- and 11.29-fold upregulations under both conditions (Fig. 3). However, most genes encoding the enzymes responsible for the metabolism of galactose, starch, sucrose, purine, and histidine, as well as those for valine and isoleucine biosynthesis, showed reduced expression in recombinant strains under normal and acid-stress conditions, which corresponded to the decreased expression of genes involved in sugar transport (*ptcA*, *malFG*, *fruA*, and *LLNZ_RS04080*). Moreover, several genes implicated in pyrimidine metabolism (*pyrCBDEK*) were up-regulated during acid stress (Fig. 3a). Interestingly, the *cspABCD2* genes and multiple transcriptional regulators were also consistently upregulated under both conditions. Meanwhile, the genes *fabH*, *busAA*, and *busAB*, which encode glycine/betaine ABC transporters, were also highly up-regulated under both conditions (Fig. 3b).

**Fig. 4:**
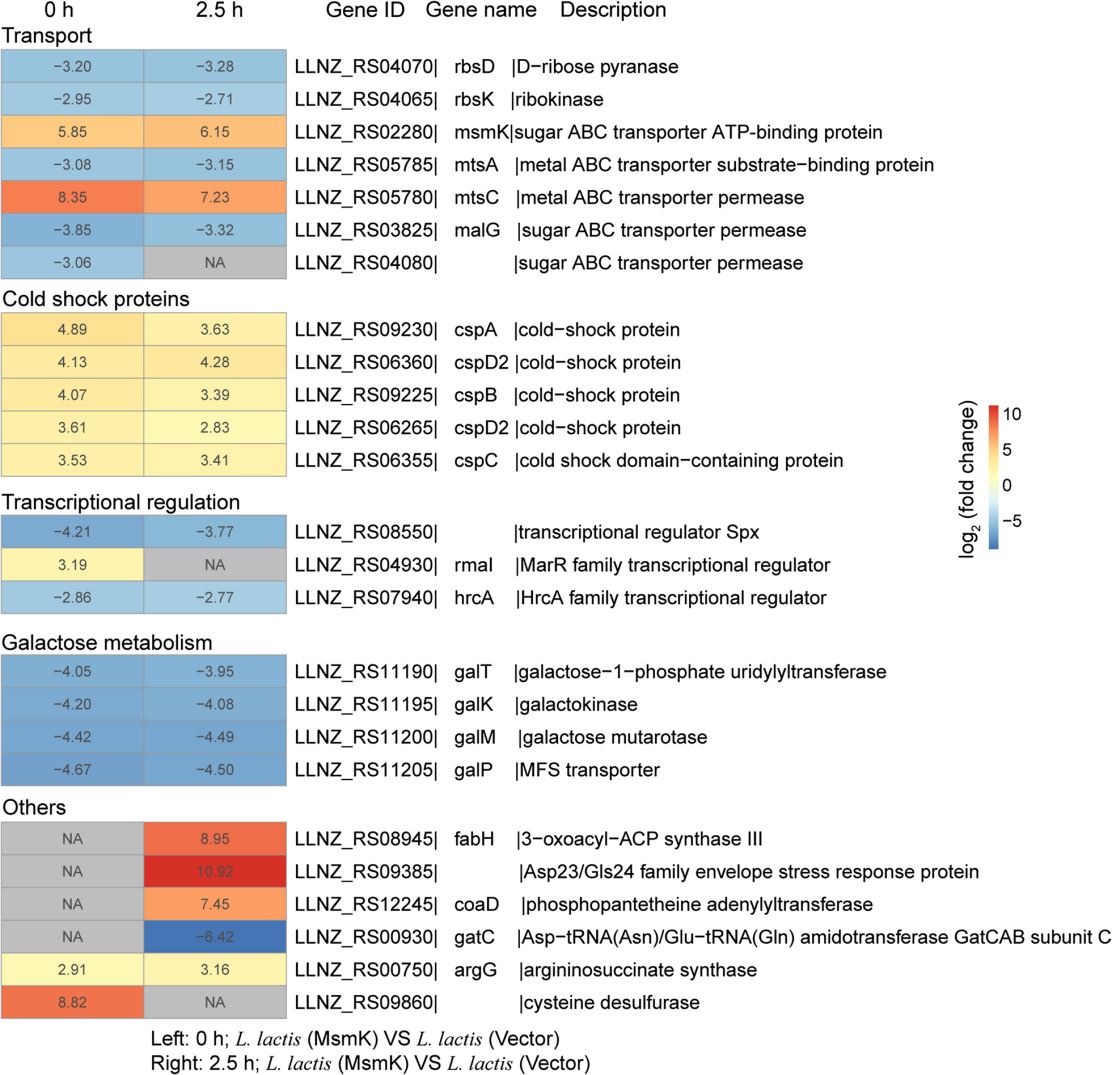
Heatmap of important differentially expressed genes in the recombinant strain (*L. lactis* (MsmK)) relative to the control strain (*L. lactis* (Vector)) under normal (0 h) and acid-stress (2.5 h) conditions. Each gene shows the expression ratio (log_2_-fold change). NA represents the expression of gene was upregulated or downregulated with a less than twofold change. Genes with at least a twofold change are shown. Adjusted p < 0.05 for all data selected

Furthermore, in *L. lactis* (MsmK), we found that in addition to the up-regulation of *cspABCD2* and the down-regulation of galactose metabolism pathway-related genes (*galKMPT*), genes related to transport (*mtsC*) and arginine biosynthesis (*argG*) were also highly up-regulated under normal and acid-stress conditions (Fig. 4). During acid stress, we also found that *fabH*, *LLNZ_RS09385*, and *coaD* genes were up-regulated in the recombinant strain.

**Fig. 5:**
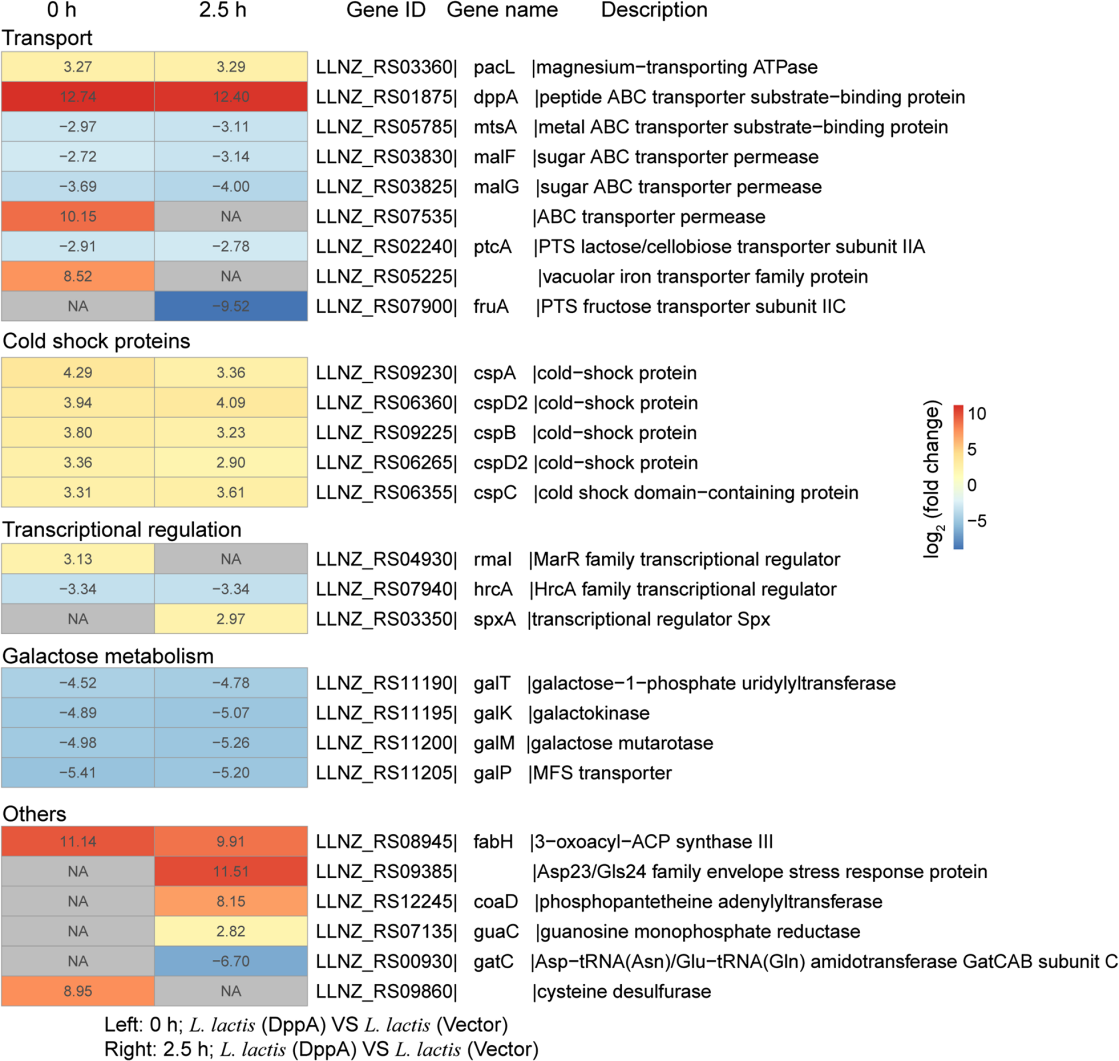
Heatmap of important differentially expressed genes in the recombinant strain (*L. lactis* (DppA)) relative to the control strain (*L. lactis* (Vector)) under normal (0 h) and acid-stress (2.5 h) conditions. Each gene shows the expression ratio (log_2_-fold change). NA represents the expression of gene was upregulated or downregulated with a less than twofold change. Genes with at least a twofold change are shown. Adjusted p < 0.05 for all data selected

Finally, we analyzed the key DEGs between the recombinant strain *L. lactis* (DppA) and the control strain *L. lactis* (Vector). In addition to the *cspABCD2* and *galKMPT* DEGs, the genes *pacL* and *fabH* were up-regulated in the recombinant strain under both conditions (Fig. 5). Among them, the *fabH* gene showed dramatic 11.14- and 9.91-fold up-regulations, respectively. Meanwhile, we found that the transcriptional regulators *rmal* and *spxA* showed identical expression patterns to those in the recombinant strain *L. lactis* (RbsA). Moreover, the genes *LLNZ_RS09385*, *coaD*, and *guaC* were also up-regulated in the recombinant strain during acid stress.

### Integrated transcriptome analysis of the four recombinant strains

Based on the key DEGs identified in the four recombinant strains, we can conclude that transport, metabolism, and transcriptional regulation were the most commonly affected processes under acid stress. Furthermore, the four overexpressed genes are all ABC family transporters, which may share some common acid-stress response mechanisms. Therefore, we further analyzed the common DEGs among the four recombinant strains compared to control strain, respectively (Additional file 1: Fig. S2). The major *csp* genes, which encode cold-shock proteins, were up-regulated in all four recombinant strains under normal and acid-stress conditions. Furthermore, the expression of *galKMPT* genes were significantly repressed under both conditions. In addition, we found that the *fabH* and *coaD* genes showed dramatic up-regulation in these recombinant strains during acid stress. Based on these results, it can be concluded that the four ABC transporters confer acid-stress tolerance to *L. lactis* through several shared response mechanisms, including regulating the expression of related genes involved in cold-shock proteins (*csp*), galactose metabolism (*galKMPT*), fatty acid biosynthesis (*fabH*), and coenzyme A (*coaD*).

### Effects of overexpressing ABC transporters on intracellular ATP concentration under acid stress

**Fig. 6:**
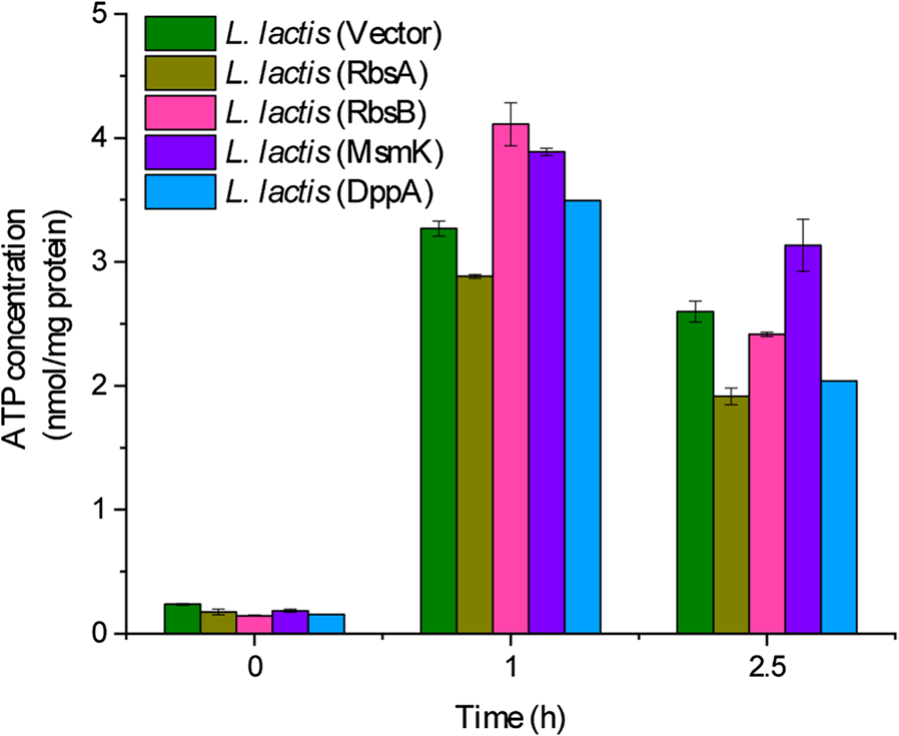
Effects of over-expressed ABC transporters on the intracellular ATP concentrations during acid stress. All strains were exposed to acid stress at pH 4.0 for various times (0, 1 and 2.5 h). Error bars represent the mean ± standard deviation of three replicates

Since most acid-stress processes require energy consumption, we further measured the intracellular ATP concentration to investigate the changes in intracellular energy production during acid stress. Time-course measurements of the intracellular ATP concentration exhibited that the recombinant strains *L. lactis* (RbsB) and *L. lactis* (MsmK) maintained a higher ATP concentration than the control strain after acid shock for 1 h at pH 4.0, which increase of 25.7% and 18.9%, respectively, compared to the control strain (Fig. 6). Thereafter, the ATP concentration began to decline gradually, and the recombinant strain *L. lactis* (MsmK) displayed higher ATP level that was 1.2-fold higher than that in the control strain after acid shock for 2.5 h. These results demonstrated that the overexpression of the ABC transporters RbsB and MsmK increased intracellular ATP concentrations to protect cells against acid stress in the initial stage of acid stress. Meanwhile, the recombinant strain *L. lactis* (MsmK) maintained elevated ATP concentrations during acid stress.

## Discussion

The ABC protein family is one of the most abundant protein superfamilies, and its members mainly mediate the transport of nutrients and other molecules into cells or the pumping of toxins and lipids across membranes. Moreover, during acid stress, microbial cells need to import more nutrients and export toxins across the membrane to protect the cells against acid stress. Therefore, in this study, we performed a detailed analysis of ABC superfamily proteins in *L. lactis* to determine their relevance to acid stress.

The ribose transporters in *L. lactis* is a complex consisting of an ATP-binding cassette protein, RbsA; a substrate binding protein, RbsB; and RbsCD. In *E. coli*, the ribose transporter is critical for the uptake of ribose, while the *rbsA* and *rbsB* genes form a part of the *rbs* operon, whose products are involved in the transmitting of molecular precursors for nucleic acid synthesis [29]. However, in *L. lactis*, it is still unclear how the ribose transporter protects cells against acid stress. Thus, we overexpressed the *rbsA* and *rbsB* genes in *L. lactis*, respectively, which their expression showed significant difference in our previous study. In addition, the *rbsA* and *rbsB* genes were also co-expressed in *L. lactis* to investigate whether acid stress tolerance could be further improved. Unfortunately, the co-expressing strains did not exhibit higher survival rates compared to single gene-expressing strains (data not shown).

In response to acid stress, the carbohydrate metabolism can be strengthened to produce more energy, and microbial cells can consume the energy to against acid stress [30]. The acquisition and metabolism of carbohydrates is essential for the survival of *L. lactis* under acid stress. However, excessive transport of carbohydrates may result in a rapid accumulation of toxic glycolysis intermediates, acidification of intracellular environment and osmotic stress [31]. Therefore, microbial cells need to adjust their metabolism and gene expression patterns to achieve optimal utilization of carbohydrates [32]. The MsmK protein is an ATPase that is responsible for the utilization of various carbohydrates. It has been shown in *Streptococcus suis* that MsmK is essential not only for the utilization of various carbohydrates, but also for successful survival and colonization [33]. Interestingly, two sugar ABC transporters (*malG*, and *LLNZ_RS04080*) were downregulated in *L. lactis* (MsmK). Therefore, we speculate that *L. lactis* may have developed a self-regulatory mechanism to achieve optimal flow of metabolism and transport of carbohydrates, and the MsmK protein may contribute to acid stress by regulating the utilization of carbohydrates during acid stress.

Peptide metabolism and transport have been widely investigated in Gram-positive bacteria. The most common peptide transporters are binding-protein-dependent transporters, which mainly includes oligopeptides (Opp), dipeptides (Dpp), and tripeptides (TPP) [34]. Among these transport systems, the Opp systems have been extensively characterized and were found to be associated with stress tolerance. The Opp systems have been found to transport various peptides and are involved in recycling the cell wall peptides for the synthesis of new peptidoglycan in some *Streptococcus* spp. [35]. In addition, the OppA protein was found to be up-regulated under acid stress in a proteomics analysis of *L. reuteri* ATCC 23272 [36]. In this work, we investigated the DppA protein, a Dpp-binding protein precursor that belongs to the Opp transport system substrate-binding protein family. However, little is known about its functional role in *L. lactis* during acid stress.

In this study, we performed transcriptome analysis in four recombinant strains to study the mechanisms underlying improved acid tolerance mediated by the ABC transporters. In addition, we also further analyzed the common DEGs among the four recombinant strains when compared to the control strain, respectively (Additional file 1: Fig. S2). Several *csp* genes were up-regulated in all four recombinant strains under normal and acid-stress conditions. The main classes of bacterial molecular chaperones include DnaK/Hsp70, GroEL/Hsp60, and the heat/cold shock proteins; and molecular chaperones are implicated in protein folding, protein renaturation or degradation under stress, protein targeting to membranes, and the control of protein–protein interactions [37]. Moreover, the binding proteins were found to interact with unfolding and denatured proteins, such as the molecular chaperones. In addition to their function in transport, binding proteins were shown to help in protein folding and protection from stress [38]. Thus, we proposed that these recombinant strains could help cells withstand acid stress by up-regulating the expression of genes encoding cold-shock proteins. In addition, the genes *fabH* and *coaD* also showed highly up-regulations in the recombinant strains during acid stress. In *L. lactis*, the process of fatty acids elongation is initiated by FabH by condensing an acetyl-CoA with malonyl-ACP [39]. The up-regulation of the *fabH* gene may improve the fluidity and permeability of cell membranes by regulating the composition of fatty acids, thereby maintaining cell homeostasis and efficient transmembrane transport processes. Moreover, the CoaD protein is one of the key enzymes of coenzyme A biosynthesis pathway, and coenzyme A is mainly involved in fatty acids and pyruvate metabolism. Thus, we may conclude that the enhancement of coenzyme A biosynthesis regulates intracellular fatty acid and pyruvate metabolism, thereby helping cells resist acid stress.

In addition to the common acid-stress-response mechanisms mediated by ABC transporters, some specific DEGs were found in individual recombinant strains. In *L. lactis* (RbsB), the genes involved in the pyrimidine biosynthetic pathway (*pyrCBDEK*) were up-regulated under acid stress (Fig. 3a). The *pyrCBDEK* genes mainly mediate in the conversion of glutamine to UMP, which can be further converted into UTP, CTP, dCTP, and dTTP. In addition, the pyrimidine biosynthetic pathway is linked to arginine biosynthesis by carbamoyl phosphate [15]. Therefore, the up-regulation of *pyrCBDEK* genes may affect the arginine biosynthesis pathway. In addition, betaine has been shown to protect cells from acid stress, and bacterial cells can improve their acid-stress tolerance by strengthening the transport of betaine (*busAA, AB*) during acid stress [40] (Fig. 3b).

Interestingly, we found that various genes encoding cell well anchor proteins were abundant. As the primary barrier for nutrients or ions entering into cells, cell well is closely related to microbial acid tolerance. Bacteria need to sustain a robust cell wall to provide optimal environment for cell growth and metabolism during acid stress. Cell wall has been found to play important roles in resisting acid stress and nisin production in *L. lactis*. Increasing O-acetylation and N-deacetylation in cell wall improved autolysis resistance by decreasing the susceptibility to PG hydrolases, and therefore contributed to cell wall integrity and the improved acid tolerance of *L. lactis* F44 [41]. In addition, the acid tolerance and nisin production could be improved by genetically increasing D-Asp amidation level in cell wall in *L. lactis* F44 [42]. In this study, the *LLNZ_RS12985* gene was downregulated in *L. lactis* (RbsA) and *L. lactis* (RbsB) during acid stress. Nevertheless, the *LLNZ_RS13320* gene showed upregulation in *L. lactis* (MsmK) and *L. lactis* (DppA) during acid stress (Additional file 2). The differential expression of these genes may contribute to cell wall integrity and help cells resist acid stress.

ABC proteins are ATP dependent membrane-bound transporters that use the binding and hydrolysis of ATP to transport a wide variety of substrates, ranging from ions to macromolecules, across membranes [43], and this process requires the hydrolysis of ATP. Therefore, we measured the intracellular ATP concentrations of the recombinant and control strains during acid stress (Fig. 6). In this work, the results indicated that intracellular ATP concentrations increased within the first 1 h of stress, then gradually decreased. This may have been caused by cell sensing in the early stages of stress, thereby allowing more ATP to be generated in response to acid stress [25]. Interestingly, we found that the recombinant strain *L. lactis* (MsmK) showed the highest survival rates than the other three strains. Meanwhile, overexpression of MsmK protein up-regulated the expression of several genes (*argG*, *coaD*) involved in pathways of energy generation (Fig. 4), and *L. lactis* (MsmK) maintained an elevated ATP concentration than the control strain during acid stress (Fig. 6). In our previous study, the ArgG protein (argininosuccinate synthase) had been found to enhance the acid tolerance of *L. lactis*. Overexpression of ArgG protein could enhance the metabolic flux of arginine deiminase (ADI) pathway, which could generate more ATP, and the recombinant strain maintained higher ATP level than control strain during acid stress [44]. Therefore, we speculate that the highest survival rate exhibited by overexpression of MsmK protein may be due in part to the up-regulated expression of *argG* gene, which was associated with elevated ATP level.

## Conclusions

An ideal cell factory should demonstrate the efficient production of targeted products, and this requires the host to maintain high metabolic activity in an acidic environment during the process of producing acidic products. In this study, the overexpression of ABC transporters was performed to enhance the acid tolerance of *L. lactis*. Here, we showed that the four overexpressing strains exhibited higher survival rates than the control strain under acid stress. Moreover, by means of comparative transcriptomics, this study elucidated the transcriptional response mechanisms of the recombinant strains during acid stress. The four recombinant strains not only share several response mechanisms, such as enhancing the expression of genes involved in cold-shock proteins (*csp*), fatty acid biosynthesis (*fabH*), and coenzyme A biosynthesis (*coaD*), but certain specific recombinant strains also showed unique acid-stress response mechanisms. This study indicates that genetic engineering through overexpression of ABC transporters is a promising strategy to improve the acid tolerance of *L. lactis*. These genetically engineered strains with improved tolerance to acid stress are promising candidates for food and industrial applications.

## Additional files

**Additional file 1: Fig. S1.** Overall differentially expressed genes during acid stress. **Fig. S2.** Heatmaps of common differentially expressed genes in recombinant strains when compared to control strain. **Table S1.** Primers used in PCR amplifications. **Table S2.** The numbers of upregulated and downregulated genes through the eight groups.

**Additional file 2.** Details of all differentially expressed genes.

## Data Availability

All data generated or analyzed in this study are included in this published article and the Additional files 1 and 2.

## References

[1] Song AA-L, In LLA, Lim SHE, Rahim RA (2017). A review on *Lactococcus lactis*: from food to factory. Microb Cell Fact.

[2] Wu CD, Huang J, Zhou RQ (2017). Genomics of lactic acid bacteria: current status and potential applications. Crit Rev Microbiol.

[3] de Ruyter PG, Kuipers OP, de Vos WM (1996). Controlled gene expression systems for *Lactococcus lactis* with the food-grade inducer nisin. Appl Environ Microbiol.

[4] van Rooijen RJ, Gasson MJ, De Vos W (1992). Characterization of the *Lactococcus lactis* lactose operon promoter: contribution of flanking sequences and LacR repressor to promoter activity. J Bacteriol.

[5] Papadimitriou K, Alegría Á, Bron PA, De Angelis M, Gobbetti M, Kleerebezem M, Lemos JA, Linares DM, Ross P, Stanton C (2016). Stress physiology of lactic acid bacteria. Microbiol Mol Biol Rev.

[6] Wu CD, Huang J, Zhou RQ (2014). Progress in engineering acid stress resistance of lactic acid bacteria. Appl Microbiol Biotechnol.

[7] Broadbent JR, Larsen RL, Deibel V, Steele JL (2010). Physiological and transcriptional response of *Lactobacillus casei* ATCC 334 to acid stress. J Bacteriol.

[8] Zhu Z, Zhang J, Ji X, Fang Z, Wu Z, Chen J, Du G (2018). Evolutionary engineering of industrial microorganisms-strategies and applications. Appl Microbiol Biotechnol.

[9] Zhang J, Wu C, Du G, Chen J (2012). Enhanced acid tolerance in *Lactobacillus casei* by adaptive evolution and compared stress response during acid stress. Biotechnol Bioprocess Eng.

[10] Patnaik R, Louie S, Gavrilovic V, Perry K, Stemmer WPC, Ryan CM, del Cardayre S (2002). Genome shuffling of *Lactobacillus* for improved acid tolerance. Nat Biotechnol.

[11] Alper H, Moxley J, Nevoigt E, Fink GR, Stephanopoulos G (2006). Engineering yeast transcription machinery for improved ethanol tolerance and production. Science.

[12] Wu C, Zhang J, Du G, Chen J (2013). Aspartate protects *Lactobacillus casei* against acid stress. Appl Microbiol Biotechnol.

[13] Gong ZW, Nielsen J, Zhou YJJ (2017). Engineering robustness of microbial cell factories. Biotechnol J.

[14] Zhai ZY, An HR, Wang GH, Luo YB, Hao YL (2015). Functional role of pyruvate kinase from *Lactobacillus bulgaricus* in acid tolerance and identification of its transcription factor by bacterial one-hybrid. Sci Rep.

[15] Wu H, Liu J, Miao S, Zhao Y, Zhu H, Qiao M, Saris PEJ, Qiao J (2018). Contribution of YthA, a PspC family transcriptional regulator to *Lactococcus lactis* F44 acid tolerance and nisin yield: a transcriptomic approach. Appl Environ Microbiol.

[16] Abdullah Al M, Sugimoto S, Higashi C, Matsumoto S, Sonomoto K (2010). Improvement of multiple-stress tolerance and lactic acid production in *Lactococcus lactis* NZ9000 under conditions of thermal stress by heterologous expression of *Escherichia coli dnaK*. Appl Environ Microbiol.

[17] Gaida SM, Al-Hinai MA, Indurthi DC, Nicolaou SA, Papoutsakis ET (2013). Synthetic tolerance: three noncoding small RNAs, DsrA, ArcZ and RprA, acting supra-additively against acid stress. Nucleic Acids Res.

[18] Gao X, Jiang L, Zhu LY, Xu Q, Xu X, Huang H (2016). Tailoring of global transcription sigma D factor by random mutagenesis to improve *Escherichia coli* tolerance towards low-pHs. J Biotechnol.

[19] Higgins CF (1992). ABC transporters: from microorganisms to man. Annu Rev Cell Biol.

[20] Prasad R, Goffeau A (2012). Yeast ATP-binding cassette transporters conferring multidrug resistance. Annu Rev Microbiol.

[21] Wang GH, Li D, Ma XY, An HR, Zhai ZY, Ren FZ, Hao YL (2015). Functional role of *oppA* encoding an oligopeptide-binding protein from *Lactobacillus salivarius* Ren in bile tolerance. J Ind Microbiol Biotechnol.

[22] Madeo M, O’Riordan N, Fuchs TM, Utratna M, Karatzas KAG, O’Byrne CP (2012). Thiamine plays a critical role in the acid tolerance of *Listeria monocytogenes*. FEMS Microbiol Lett.

[23] Zhang MM, Zhang KY, Mehmood MA, Zhao ZK, Bai FW, Zhao XQ (2017). Deletion of acetate transporter gene *ADY2* improved tolerance of *Saccharomyces cerevisiae* against multiple stresses and enhanced ethanol production in the presence of acetic acid. Bioresour Technol.

[24] Holo H, Nes IF (1989). High-frequency transformation, by electroporation, of *Lactococcus lactis* subsp. cremoris grown with glycine in osmotically stabilized media. Appl Environ Microbiol.

[25] Zhu Z, Ji X, Wu Z, Zhang J, Du G (2018). Improved acid-stress tolerance of *Lactococcus lactis* NZ9000 and *Escherichia coli* BL21 by overexpression of the anti-acid component *recT*. J Ind Microbiol Biotechnol.

[26] Kim D, Langmead B, Salzberg SL (2015). HISAT: a fast spliced aligner with low memory requirements. Nat Methods.

[27] Pertea M, Pertea GM, Antonescu CM, Chang T-C, Mendell JT, Salzberg SL (2015). StringTie enables improved reconstruction of a transcriptome from RNA-seq reads. Nat Biotechnol.

[28] Wang L, Feng Z, Wang X, Wang X, Zhang X (2010). DEGseq: an R package for identifying differentially expressed genes from RNA-seq data. Bioinformatics.

[29] Barroga CF, Zhang H, Wajih N, Bouyer JH, Hermodson MA (1996). The proteins encoded by the rbs operon of *Escherichia coli*: I. Overproduction, purification, characterization, and functional analysis of RbsA. Protein Sci.

[30] Zhang J, Caiyin Q, Feng WJ, Zhao XL, Qiao B, Zhao GR, Qiao JJ (2016). Enhance nisin yield via improving acid-tolerant capability of *Lactococcus lactis* F44. Sci Rep.

[31] Lemos JA, Burne RA (2008). A model of efficiency: stress tolerance by *Streptococcus mutans*. Microbiology.

[32] Lemos JA, Nascimento MM, Lin VK, Abranches J, Burne RA (2008). Global regulation by (p) ppGpp and CodY in *Streptococcus mutans*. J Bacteriol.

[33] Tan M-F, Gao T, Liu W-Q, Zhang C-Y, Yang X, Zhu J-W, Teng M-Y, Li L, Zhou R (2015). MsmK, an ATPase, contributes to utilization of multiple carbohydrates and host colonization of *Streptococcus suis*. PLoS ONE.

[34] Borezee E, Pellegrini E, Berche P (2000). OppA of *Listeria monocytogenes*, an oligopeptide-binding protein required for bacterial growth at low temperature and involved in intracellular survival. Infect Immun.

[35] Goodell EW, Higgins CF (1987). Uptake of cell wall peptides by *Salmonella typhimurium* and *Escherichia coli*. J Bacteriol.

[36] Lee K, Lee HG, Pi K, Choi YJ (2008). The effect of low pH on protein expression by the probiotic bacterium *Lactobacillus reuteri*. Proteomics.

[37] Feder ME, Hofmann GE (1999). Heat-shock proteins, molecular chaperones, and the stress response: evolutionary and ecological physiology. Annu Rev Physiol.

[38] Richarme G, Caldas TD (1997). Chaperone properties of the bacterial periplasmic substrate-binding proteins. J Biol Chem.

[39] Eckhardt TH, Skotnicka D, Kok J, Kuipers OP (2013). Transcriptional regulation of fatty acid biosynthesis in *Lactococcus lactis*. J Bacteriol.

[40] Sheehan VM, Sleator RD, Hill C, Fitzgerald GF (2007). Improving gastric transit, gastrointestinal persistence and therapeutic efficacy of the probiotic strain *Bifidobacterium breve* UCC2003. Microbiology.

[41] Cao L, Liang D, Hao P, Song Q, Xue E, Caiyin Q, Cheng Z, Qiao J (2018). The increase of O-acetylation and N-deacetylation in cell wall promotes acid resistance and nisin production through improving cell wall integrity in *Lactococcus lactis*. J Ind Microbiol Biotechnol..

[42] Hao P, Liang D, Cao L, Qiao B, Wu H, Caiyin Q, Zhu H, Qiao J (2017). Promoting acid resistance and nisin yield of *Lactococcus lactis* F44 by genetically increasing D-Asp amidation level inside cell wall. Appl Microbiol Biotechnol.

[43] Rees DC, Johnson E, Lewinson O (2009). ABC transporters: the power to change. Nat Rev Mol Cell Biol.

[44] Zhang M, Zhang J, Liu L, Du G, Chen J (2017). Influence of key acid-resistant genes in arginine metabolism on stress tolerance in *Lactococcus lactis* NZ9000. Microbiol China.

[45] Kuipers OP, de Ruyter PGGA, Kleerebezem M, de Vos WM (1998). Quorum sensing-controlled gene expression in lactic acid bacteria. J Biotechnol.

[46] Casadaban MJ, Cohen SN (1980). Analysis of gene control signals by DNA fusion and cloning in *Escherichia coli*. J Mol Biol.

[47] Li Y, Hugenholtz J, Sybesma W, Abee T, Molenaar D (2005). Using *Lactococcus lactis* for glutathione overproduction. Appl Microbiol Biotechnol.

