# Supplementary material for: Enhanced acid-stress tolerance in Lactococcus lactis NZ9000 by overexpression of ABC transporters
Source: Microb Cell Fact. 2019 Aug 13;18:136. doi: 10.1186/s12934-019-1188-8 (PMC6693162; doi:10.1186/s12934-019-1188-8)
Supplement: Supplementary file 1 — Additional file 1: Fig. S1. Overall differentially expressed genes during acid stress. Fig. S2. Heatmaps of common differentially expressed genes in recombinant strains when compared to control strain. Table S1. Primers used in PCR amplifications. Table S2. The numbers of upregulated and downregulated genes through the eight groups. [file 12934_2019_1188_MOESM1_ESM.docx]

**Enhanced acid-stress tolerance in *Lactococcus lactis* NZ9000 by overexpression of ABC transporters**

Zhengming Zhu^1,3^, Jinhua Yang^1,3^, Peishan Yang^1,3^, Zhimeng Wu^2,3^, Juan Zhang^1,3^*, Guocheng Du^2,3^

^1^ Key Laboratory of Industrial Biotechnology, Ministry of Education, School of Biotechnology, Jiangnan University, 1800 Lihu Road, Wuxi, Jiangsu 214122, China;

^2^ The Key Laboratory of Carbohydrate Chemistry and Biotechnology, Ministry of Education, Jiangnan University, 1800 Lihu Road, Wuxi, Jiangsu 214122, China;

^3^ School of Biotechnology, Jiangnan University, 1800 Lihu Road, Wuxi, Jiangsu 214122, China;

^*^ Corresponding authors

Mailing address: School of Biotechnology, Jiangnan University, 1800 Lihu Road, Wuxi, Jiangsu 214122, China.

Phone: +86-510-85918307, Fax: +86-510-85918309

E-mail: [zhangj@jiangnan.edu.cn](mailto:zhangj@jiangnan.edu.cn)


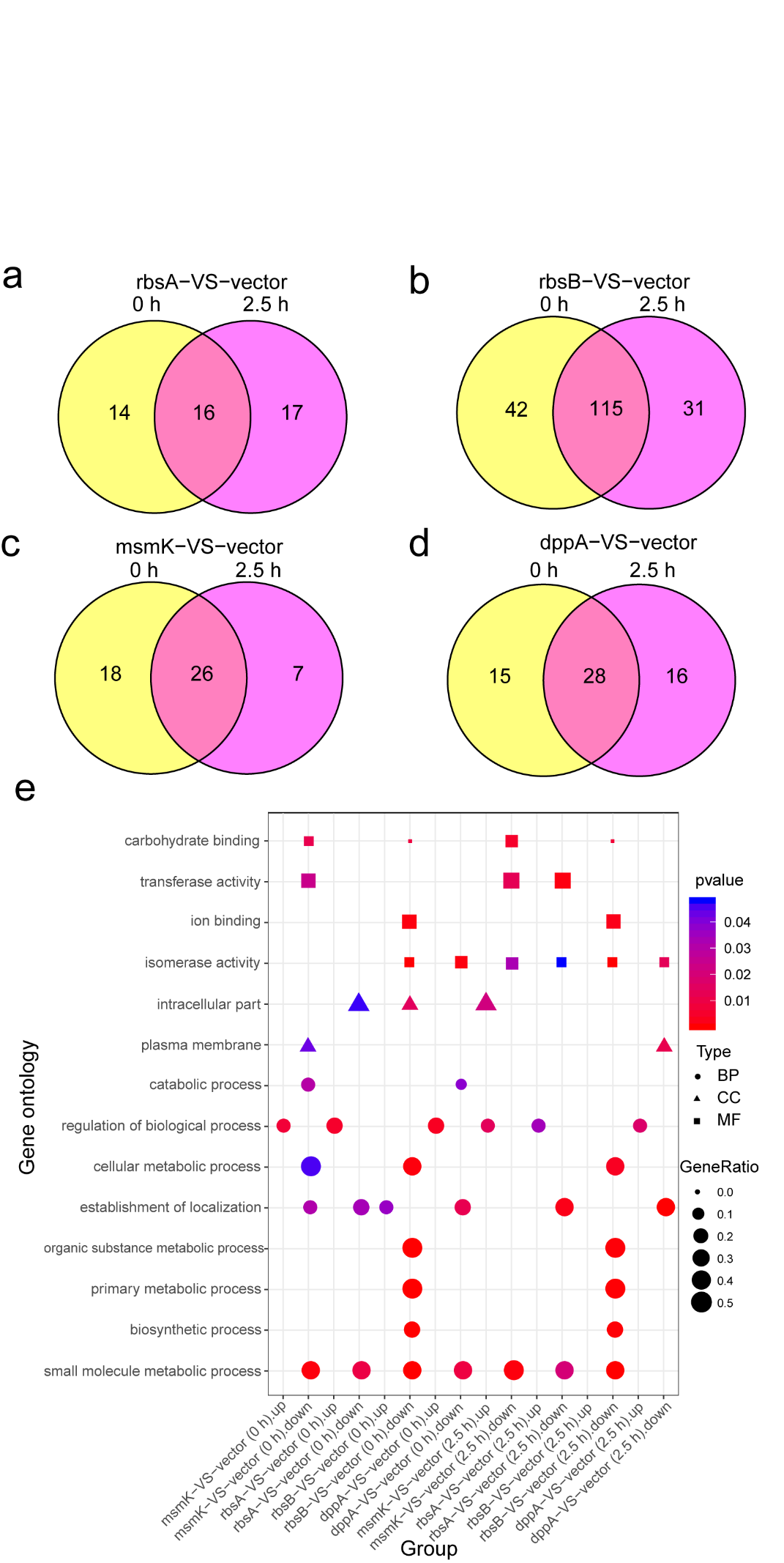


Fig. S1: Overall differentially expressed genes during acid stress. The Venn diagrams depict the number of differentially expressed genes under normal (0 h) and acid-stress (2.5 h) conditions in the recombinant (*L. lactis* (RbsA) (a), *L. lactis* (RbsB) (b), *L. lactis* (MsmK) (c), *L. lactis* (DppA) (d)) and control (*L. lactis* (Vector)) strains. (e) Gene ontology analysis of the differentially expressed genes. Genes with at least a 2-fold change are shown. Adjusted p < 0.05 for all data selected.


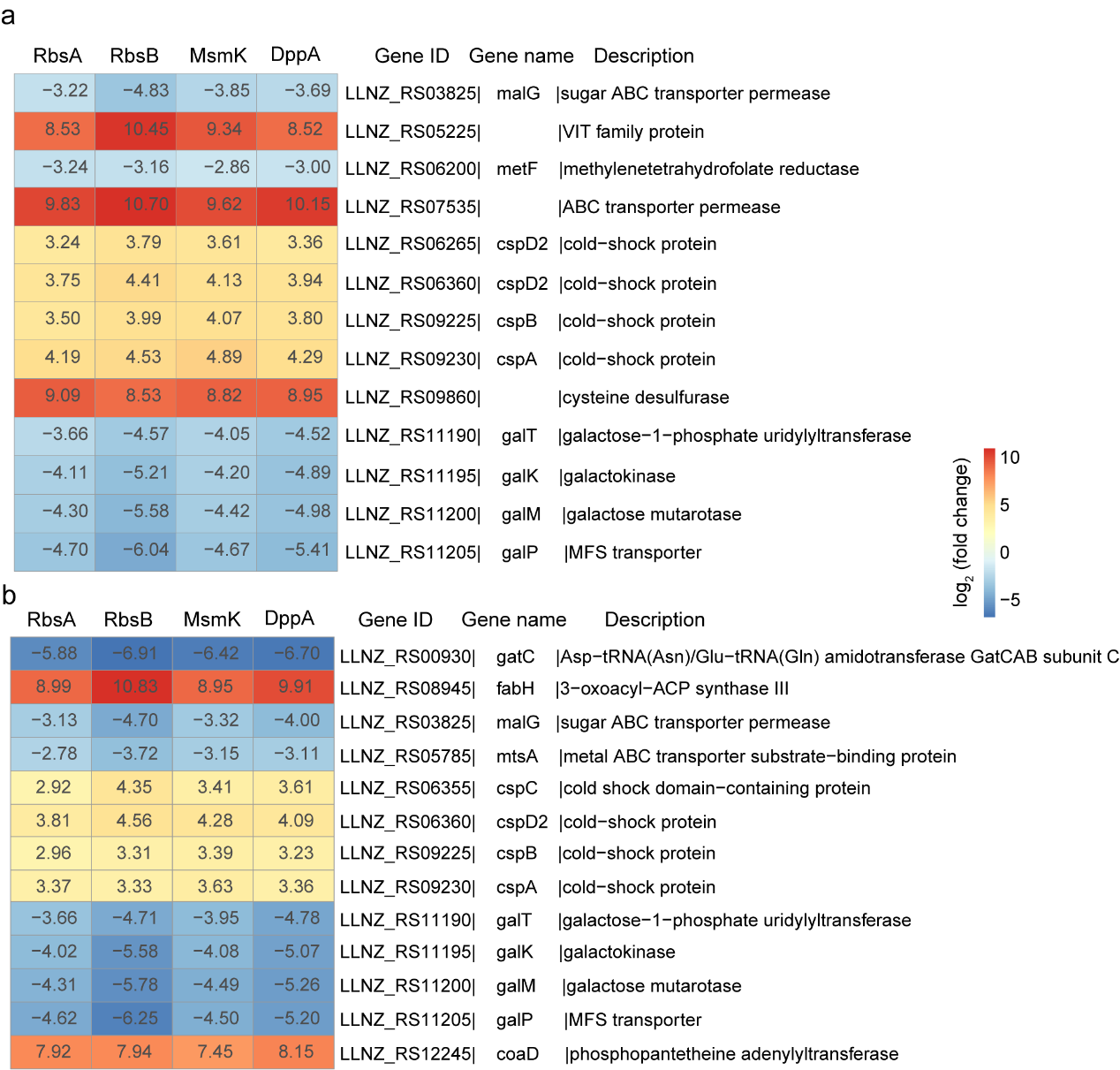


Fig. S2: Heatmaps of common differentially expressed genes in recombinant strains when compared to control strain. (a) normal condition (0 h), (b) acid-stress condition (2.5 h). Genes with at least a 2-fold change are shown. Adjusted p < 0.05 for all data selected.

**Table S1.** Primers used in PCR amplifications

| Primers | Sequence (from 5´ to 3´) ^a^ | Restriction site | Products |
| --- | --- | --- | --- |
| pNZ8148/rbsA-F | CATGCCATGGGGTTGAAAATAGAAATGAAGAACATTTCTAAATC | *Nco*I | pNZ8148/RbsA |
| pNZ8148/rbsA-R | CCAAGCTTTCATTTTCCTCCTGTTGCGAG | *Hin*dIII |  |
| pNZ8148/rbsB-F | CATGCCATGGGGATGAAATTAGTAAAAAAATTAACTTTTGCC | *Nco*I | pNZ8148/RbsB |
| pNZ8148/rbsB-R | CCAAGCTTTTACCATTTGTGTTCATCAACATTATC | *Hin*dIII |  |
| pNZ8148/msmK-F | CATGCCATGGGGATGACAACACTCGTTTTAGACAAAAT | *Nco*I | pNZ8148/MsmK |
| pNZ8148/msmK-R | GCTCTAGATTAATCAACGATACGGTGTTCAGT | *Xba*I |  |
| pNZ8148/dppA-F | CATGCCATGGGGATGAAACAAGCAAAAATTATTGGG | *Nco*I | pNZ8148/DppA |
| pNZ8148/dppA-R | GCTCTAGATTATTTAATATAAGCCGATTTTAAGTCG | *Xba*I |  |

^a^Underlined bases represent enzyme restriction sites.

**Table S2.** The numbers of upregulated and downregulated genes through the eight groups.

| **NO.** | **Groups** | **upregulated**  **genes** | **downregulated**  **genes** |
| --- | --- | --- | --- |
| (1) | *L. lactis* (RbsA) -VS-*L. lactis* (Vector) (0 h) | 16 | 14 |
| (2) | *L. lactis* (RbsA) -VS-*L. lactis* (Vector) (2.5 h) | 17 | 16 |
| (3) | *L. lactis* (RbsB) -VS-*L. lactis* (Vector) (0 h) | 57 | 100 |
| (4) | *L. lactis* (RbsB) -VS-*L. lactis* (Vector) (2.5 h) | 52 | 94 |
| (5) | *L. lactis* (MsmK) -VS-*L. lactis* (Vector) (0 h) | 24 | 20 |
| (6) | *L. lactis* (MsmK) -VS-*L. lactis* (Vector) (2.5 h) | 20 | 13 |
| (7) | *L. lactis* (DppA) -VS-*L. lactis* (Vector) (0 h) | 21 | 22 |
| (8) | *L. lactis* (DppA) -VS-*L. lactis* (Vector) (2.5 h) | 21 | 23 |
